# Stochastic response analysis for nonlinear vibration systems with adjustable stiffness property under random excitation

**DOI:** 10.1371/journal.pone.0200922

**Published:** 2018-08-03

**Authors:** Shenlong Wang, Kaixin Han

**Affiliations:** School of Mechanical Engineering, University of Shanghai for Science and Technology, Shanghai, P. R. China; Vilnius University, LITHUANIA

## Abstract

Nonlinear vibration systems with adjustable stiffness property have attracted considerable attentions for their prominent broadband performances. In the present manuscript, we consider the stochastic dynamical systems with adjustable stiffness and proposed a numerical method for the random responses analysis of the Gaussian white noise excited systems. A multi-dimensional Fokker-Plank-Kolmogorov equation governing the joint probability density of the mechanical states is derived according to the theory of diffusion processes. We solve the multi-dimensional equation using a splitting method and obtained the stationary probability densities and the mean-square responses directly. Two classical nonlinear vibration systems with adjustable stiffness, including the energy harvesting system and the Duffing system with Dahl friction, are presented as examples. Their comparisons with the results from Monte-Carlo simulations illustrate the effectiveness of the proposed procedure for both monostable and bistable cases, even for cases with strong excitation. In addition, the splitting method is efficient for higher-dimensional problem and has advantages of simple implementation, less storage of intermediate values and so on. Hence, in terms of the application scope, the proposed procedure is superior to the current mainstream methods for the random response evaluation of nonlinear vibration systems with adjustable stiffness.

## 1. Introduction

Nonlinear systems with noise have attracted considerable attentions over the last few decades. In engineering applications, the vibration systems such as vibration friction systems, energy harvesting systems and vibration suppression systems are all under noise excitation. Meanwhile, the nonlinear vibration systems under random excitation are also utilized in fault diagnosis, parameter identification and structure optimization. Furthermore, those noise-excited systems are generally called stochastic systems [1–5], which are widely studied in response [6–19], stability [20–24], reliability [25–27] and so on. Among these studies, the stochastic response evaluation is the most common one.

In order to master the stochastic response property and to figure out the features of the vibration structure, such as the probability density functions (PDFs) and the mean square responses of nonlinear systems for random excitation, most researches focused on the computation method for the random vibration systems. For example, F. Rudinger [6] utilized the equivalent nonlinearization method to obtain the frequency response function and power spectral density function of the response for nonlinear systems under white noise excitation. Xu et al. [7] used the same approach to investigate the random vibration with inelastic impact subject to Gaussian white noise, so as to analytically obtain the joint probability density of nonlinear system and the statistics of system response. Jin et al. [8] also employed the equivalent nonlinearization technique and imported the generalized harmonic transformation to establish a semi-analytical solution of random response for nonlinear vibration energy harvesters subjected to Gaussian white noise excitation. One of the benefits of this approach was the applicability to the strongly nonlinear, self-excited or parametric systems compared with the equivalent linearization technique. The path-integral method showed its advantages in the computational efficiency, especially for the low-dimensional problems in engineering practice. Hence, Dimentberg et al. [9] applied the path integration method to study the SDOF stochastic vibroimpact problems and acquired the PDFs of the responses. Narayanan and Kumar [10] combined the modified path integration and the finite difference method to solve the Fokker-Planck equation of nonlinear systems subjected to random and harmonic excitations. Zhu and Duan [11] utilized the same method to research the PDFs of the non-linear ship rolling issues. Considering both the richness of the theory and the accuracy of the solution, the stochastic averaging method had prominent position in the stochastic response evaluation. Thus, Xu et al. [12] employed the stochastic averaging method to study the probability density and statistics for random responses of vibro-impact systems with inelastic contact. Gu and Zhu [13] also analyzed the random responses of the vibro-impact systems under Gaussian white noise by using the stochastic averaging method for quasi-Hamiltonian system. Anh and Hieu [14] obtained the response of the Duffing oscillator under combined periodic and random excitations by combining the stochastic averaging method, the equivalent linearization and the finite element method. Jin et al. [15] investigated the PDFs for transient responses of non-linear stochastic systems through the stochastic averaging and Mellin transform. Yang et al. [16] applied the stochastic averaging method for energy envelope to study the PDFs of stationary responses for a vibroimpact Duffing system with bilateral barriers under external and parametric Gaussian white noises. Jiang and Chen [17] utilized the stochastic averaging based on generalized harmonic functions to evaluate the responses of the energy harvesting systems under Gaussian white excitations. In addition, many other effective methods have been proposed. For instance, Rong et al. [18] used the method of multiple scales to investigate the resonant resonance response of a non-linear vibro-impact oscillator subjected to combined deterministic harmonic and random excitations. Yang et al. [19] employed the perturbation method to analyze the stationary responses of Rayleigh vibroimpact oscillator under parametric Poisson white noise.

From the investigations and results in previous studies, the equivalent nonlinearization technique [6–8] and the stochastic averaging method [12–17] have been widely applied in the random response evaluation due to their accuracy and simplicity. However, both of them have disadvantages that the approximate equivalent exists and is required necessarily, which may influence the applicability of these methods and the accuracy of the results. As an illustration, the stochastic averaging method can be only adopted to predict the random responses of light damping systems subjected to weak excitations. In order to overcome these shortcomings, we apply the splitting method [27–30] based on the Fokker-Plank-Kolmogorov equation of the original system in this paper. The method is a technique for separating multi-dimensional spatial operators into a sum of one-dimensional operators which is simple to implement, of less intermediate values storage, and flexible with respect to different boundary conditions. Furthermore, it can be efficiently and straightforwardly extended to higher-dimensional, strongly nonlinear, and strong excitation problems.

In the present manuscript, the stochastic responses of the nonlinear vibration systems with adjustable stiffness property for random excitation are investigated using the splitting method. Firstly, we derive a multi-dimensional Fokker-Plank-Kolmogorov equation governing the joint probability densities of the nonlinear systems according to the theory of diffusion processes. Secondly, we utilize a splitting method to solve the multi-dimensional equation and directly obtain the mean-square responses by the joint probability densities. Two classical nonlinear systems with variable stiffness are presented as examples. One is the energy harvesting system, and the other one is the Duffing system with Dahl friction. Finally, we compare the results obtained by splitting method with those from Monte-Carlo simulations (MCS) and evaluate the effectiveness and the applicability of the proposed procedure for both mono-stable and bi-stable cases.

## 2. Stochastic responses of adjustable stiffness systems

Consider a nonlinear vibration system with adjustable stiffness described by the following stochastic differential equations,

$$\begin{array}{l} \ddot{X}+2\xi \dot{X}+f(X)+\alpha Z=W\left(t\right)\\ \dot{Z}+\beta Z=\dot{X} \end{array}$$ (1)

in which, *X* represents the relative displacement, 2*ξ* is the viscous damping coefficient, *f*(*X*) denotes the nonlinear adjustable stiffness determining whether the system is mono-stable, bi-stable, or otherwise (subsequent details will be given in section 3), *α* and *β* are the non-dimensional parameters, *W*(*t*) is Gaussian white noise in the sense of Stratonovich with zero mean and correlation function *R*(*t*_2_−*t*_1_) = 2*Dδ*(*t*_2_−*t*_1_), in which *δ*(•) is the Dirac delta function and 2*D* is the noise intensity.

Eq (1) can be written as Itô stochastic differential equations,

$$\begin{array}{l} dX=Ydt\\ dY=-[2\xi Y+f(X)+\alpha Z]dt+\sqrt{2D}dB(t)\\ dZ=(-\beta Z+Y)dt \end{array}$$ (2)

where *B*(*t*) is an unit Wiener process.

Based on the theory of Markov process [31], the transition probability density *p*(*x*,*y*,*z*,*t*|*x*_0_,*y*_0_,*z*_0_,*t*_0_) is governed by the Fokker-Planck-Kolmogorov (FPK) equation corresponding to Itô Eq (2), given by

$$\frac{\partial p}{\partial t}=-\frac{\partial (a_{1}p)}{\partial x}-\frac{\partial (a_{2}p)}{\partial y}+\frac{1}{2}\frac{\partial^{2}(b_{22}p)}{\partial y^{2}}-\frac{\partial (a_{3}p)}{\partial z}$$ (3)

where

$$\begin{array}{l} a_{1}=y\\ a_{2}=-[2\xi y+f(x)+\alpha z],\;b_{22}=2D\\ a_{3}=-\beta z+y \end{array}$$ (4)

The initial condition for Eq (3) is

$$p(x,y,z,t|x_{0},y_{0},z_{0},t_{0})=\delta (x-x_{0},y-y_{0},z-z_{0}),t=t_{0}$$ (5)

and the boundary condition is

$$p(x,y,z,t|x_{0},y_{0},z_{0},t_{0})=0,\;(x,y,z)\in s$$ (6)

where *s* is the boundary surface.

The partial differential Eq (3) with the initial and boundary conditions can be solved by using splitting method (see S1 File) which is a technique for separating multi-dimensional spatial operators into a number of one-dimensional operators. According to the method, we obtain the following separating operators and difference equation,

$$\begin{array}{l} \frac{p^{m+1/3}-p^{m}}{\Delta t}=-\frac{\partial }{\partial x}(a_{1}\frac{p^{m+1/3}+p^{m}}{2})\\ \frac{p^{m+2/3}-p^{m+1/3}}{\Delta t}=-\frac{\partial }{\partial y}(a_{2}\frac{p^{m+2/3}+p^{m+1/3}}{2})+\frac{1}{2}\frac{\partial^{2}}{\partial y^{2}}(b_{22}\frac{p^{m+2/3}+p^{m+1/3}}{2})\\ \frac{p^{m+1}-p^{m+2/3}}{\Delta t}=-\frac{\partial }{\partial z}(a_{3}\frac{p^{m+1}+p^{m+2/3}}{2}) \end{array}$$ (7)

in which, *p*^*m*^, *p*^*m+*1/3^, *p*^*m*+2/3^, and *p*^*m+*1^ are values of function *p*(*x*,*y*,*z*,*t*|*x*_0_,*y*_0_,*z*_0_,*t*_0_) at *t* = *m*Δ*t*, (*m+*1/3)Δ*t*, (*m+*2/3)Δ*t*, and (*m+*1)Δ*t*, respectively. Eq (7) can be further written in finite difference form with respect to space, and solved by using chasing technique (see S2 File). We acquire the stationary probability density *p*(*x*,*y*,*z*) while *t* → *t*_*n*_, in which *t*_*n*_ is a relatively large number. Thus, the stochastic response of the nonlinear system with adjustable stiffness property, including *p*(*x*,*y*),*p*(*x*),*p*(*y*),*E*(*X*^2^),*E*(*Z*^2^) and so forth, are obtained by

$$\begin{array}{l} p(x,y)=\int_{-\infty }^{\infty }p(x,y,z)dz,\;p(x)=\int_{-\infty }^{\infty }\int_{-\infty }^{\infty }p(x,y,z)dydz,\\ p(y)=\int_{-\infty }^{\infty }\int_{-\infty }^{\infty }p(x,y,z)dxdz,\;p(z)=\int_{-\infty }^{\infty }\int_{-\infty }^{\infty }p(x,y,z)dxdy,\\ E(X^{2})=\int_{-\infty }^{\infty }x^{2}p(x)dx,\;E(Z^{2})=\int_{-\infty }^{\infty }z^{2}p(z)dz \end{array}$$ (8)

in which, *p*(*x*,*y*) is the joint probability density, *p*(*x*) and *p*(*y*) are the marginal probability densities, *E*(*X*^2^) and *E*(*Z*^2^) are the mean square values of random responses.

## 3. Examples and results

### 3.1. Example 1: Vibration energy harvester

**Fig 1:**
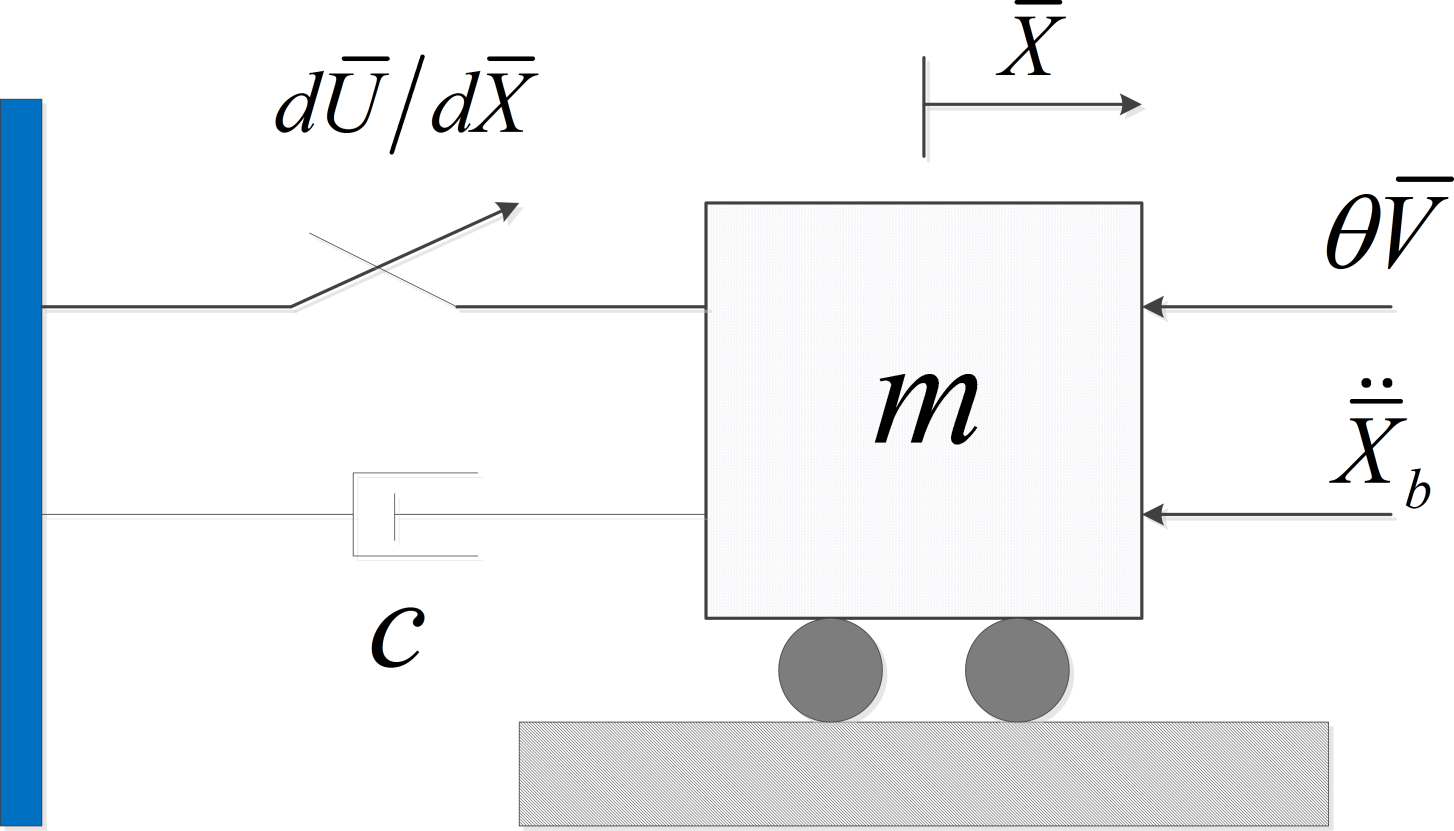
The schematic of the nonlinear piezoelectric vibration energy harvesting system.

The first example considers the model of a generic piezoelectric vibration energy harvester [8,32–35] to be simplified as a base-excited one-degree-of-freedom system coupled to a capacitive energy harvesting circuit, as shown in Fig 1.

The coupled equation governing the mechanical states and the voltage can be written as

$$\begin{array}{l} m\ddot{\bar{X}}+c\dot{\bar{X}}+\frac{d\bar{U}\left(\bar{X}\right)}{d\bar{X}}+\theta \bar{V}=-m{\ddot{\bar{X}}}_{b},\\ C_{p}\dot{\bar{V}}+\frac{\bar{V}}{R}=\theta \dot{\bar{X}} \end{array}$$ (9)

where $\bar{X}$ represents the relative displacement of mass *m* and $\bar{V}$ is the voltage measured across the equivalent load resistance *R*. *c* is the linear viscous damping coefficient, *θ* is the linear electromechanical coupling coefficient, and *C*_*p*_ is the piezoelectric capacitance. ${\ddot{\bar{X}}}_{b}$ is the base acceleration, and the dot denotes the derivative with respect to time *τ*. The function $\bar{U}\left(\bar{x}\right)$ represents the potential energy of the mechanical subsystem of the form

$$\bar{U}\left(\bar{x}\right)=\frac{1}{2}k_{1}\left(1-r\right){\bar{x}}^{2}+\frac{1}{4}k_{3}{\bar{x}}^{4}$$ (10)

in which, *k*_1_ > 0 and *k*_3_ > 0 are the linear and nonlinear stiffness coefficients, respectively, and *r* is an adjustable parameter of linear stiffness.

The coupled equations can be further non-dimensionalized by introducing the following non-dimensional parameters,

$$\begin{array}{l} X=\frac{\bar{X}}{l_{c}},\;V=\frac{C_{p}}{\theta l_{c}}\bar{V},\;t=\omega_{1}\tau ,\;\omega_{1}=\sqrt{\frac{k_{1}}{m}},\;\xi =\frac{c}{2\sqrt{k_{1}m}},\\ \kappa^{2}=\frac{\theta^{2}}{C_{p}k_{1}},\;\delta_{3}=\frac{k_{3}l_{c}^{2}}{k_{1}},\;\alpha =\frac{1}{RC_{p}\omega_{1}} \end{array}$$ (11)

where *l*_*c*_ is a length scale, *ω*_1_ represents the fundamental frequency of the degenerated linear mechanical subsystem with *k*_3_ = 0 and *r =* 0. With these transformations, we express the non-dimensional coupled equations as

$$\begin{array}{l} \ddot{X}+2\xi \dot{X}+\frac{dU\left(X\right)}{dX}+\kappa^{2}V=-{\ddot{X}}_{b},\\ \dot{V}+\alpha V=\dot{X} \end{array}$$ (12)

with

$$U\left(X\right)=\frac{1}{2}\left(1-r\right)X^{2}+\frac{1}{4}\delta_{3}X^{4}$$ (13)

In this manuscript, the base acceleration excitation ${\ddot{X}}_{b}$ is assumed as Gaussian white noise which is defined same as *W*(*t*) in Eq (1). The output power $\bar{P}={\bar{V}}^{2}/R$ is the most important quantity in energy harvesting. By introducing the transformation $P=\bar{P}/(k_{1}\omega_{1}l_{c}^{2})$, we derive the non-dimensional expression of the output power as

$$P=\kappa^{2}\alpha V^{2}$$ (14)

**Fig 2:**
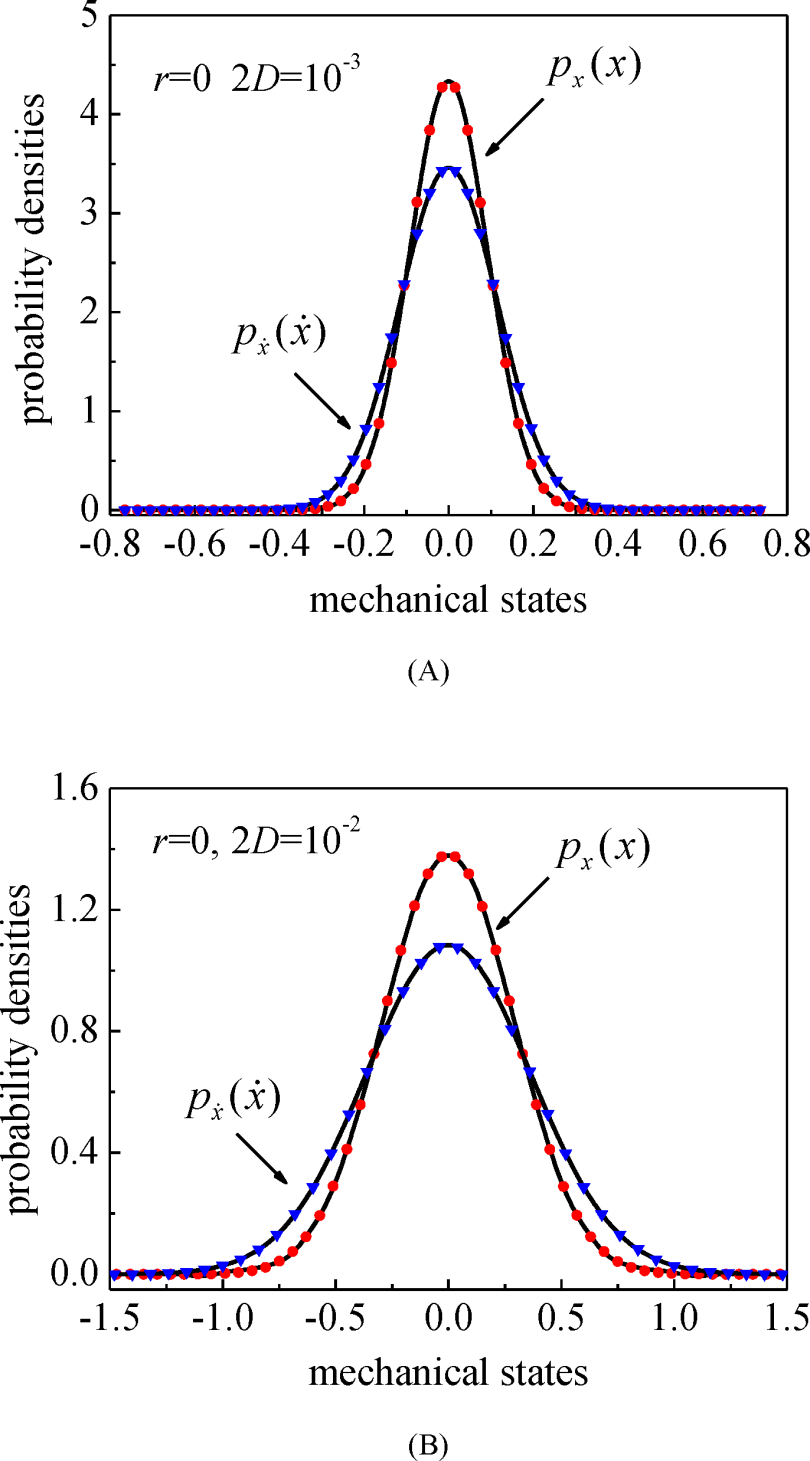
The stationary probability densities of system displacement and velocity. (A) The excitation intensity 2*D* = 10^−3^; (B) 2*D* = 10^−2^. Solid lines: the proposed numerical results; symbols: MCS results.

The parameters of the energy harvesting system (12) are set according to the research of Daqaq [36], the adjustable linear stiffness coefficient *r =* 0 for mono-stable system and *r =* 1.1 for bi-stable system, the nonlinear stiffness parameter *δ*_3_ = 0.5, the time constant ratio *α* = 0.05, the linear non-dimensional electro-mechanical coupling coefficient *κ* = 0.75, the mechanical damping ratio *ξ* = 0.01, except as specially provided. Set the time step Δ*t =* 0.02 and the calculating time *T =* 300 (see S3 File), we obtain the stationary probability densities of mono-stable system displacement and velocity with different excitation intensity and show them in Fig 2. Obviously, as the excitation intensity 2*D* changed, the responses for mono-stable system differ in magnitude but not in trend. The Monte-Carlo simulations (MCS) are carried out to evaluate the accuracy of the proposed procedure, and the results from MCS are represented by the symbols, which also applies for the figures below. It is obvious that the numerical solutions agree very well with the results from MCS.

**Fig 3:**
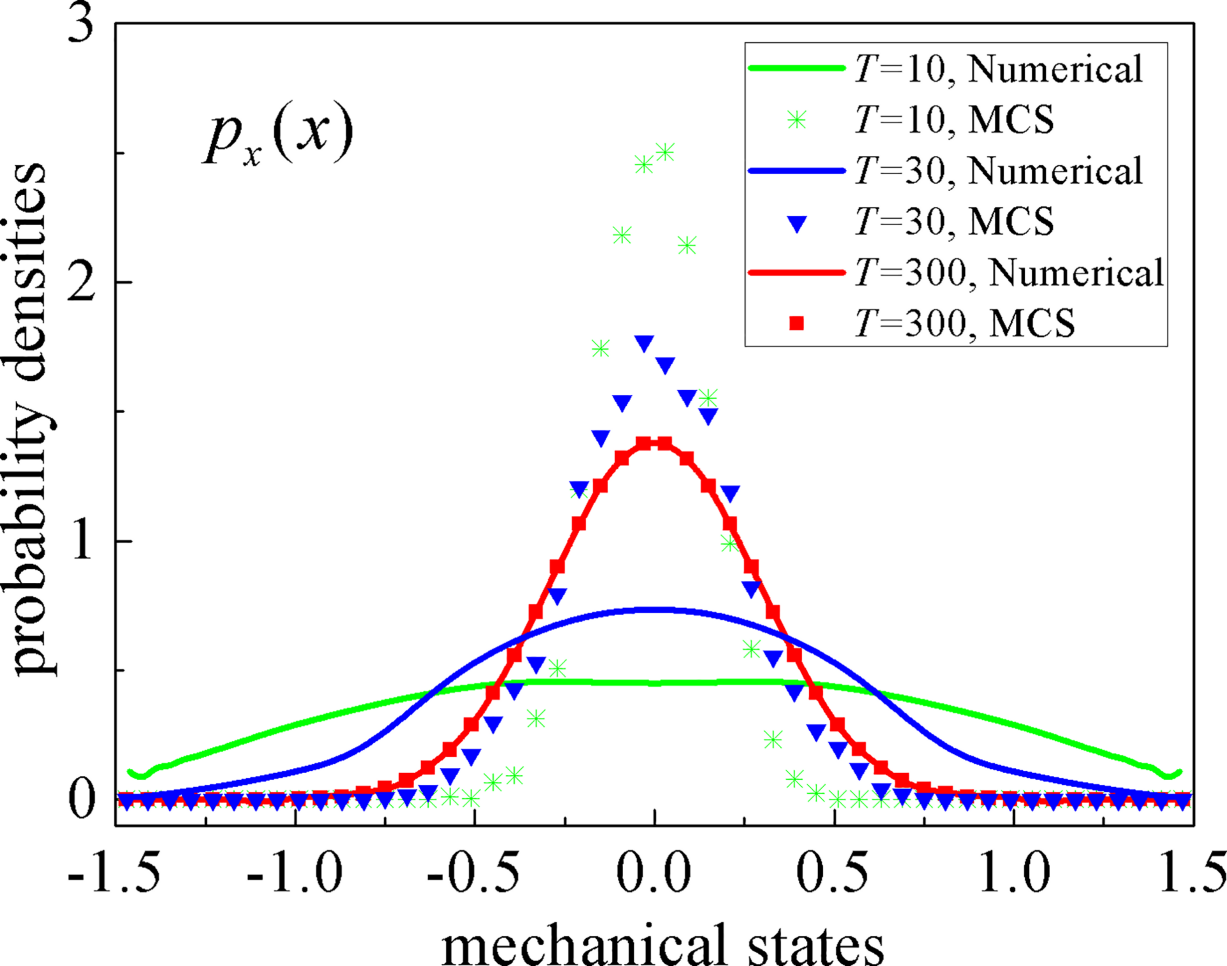
The displacement probability densities of different calculating time *T*. Solid lines: the proposed numerical results; symbols: MCS results.

We compare the stochastic responses of *T* = 10, *T* = 30, *T* = 300 and show them in Fig 3. In which, *T* = 10 and *T* = 30 correspond to transient probability densities of system displacement, while *T* = 300 correspond to stationary probability density of system displacement. It is easily seen that the splitting method is an ideal approach solving the stationary PDFs of stochastic systems but not appropriate for the transient response evaluation. We explain this phenomenon as follows: for the stationary response, terms on the l.h.s. of Eq (7) are equal to zero as there is no time variation in a stationary PDF, while (*p*^(*m*+1/3)^ - *p*^(*m*)^)/2 = (*p*^(*m*+2/3)^ - *p*^(*m*+1/3)^)/2 = (*p*^(*m*+1)^ - *p*^(*m*+2/3)^)/2 = *p*, being *p* the stationary PDF; therefore, the three equations in (7) correspond to assume that the space derivatives with respect to *X*, *Y* and *Z* are separately equal to zero, instead of being equal to zero their summation according to the FPK Eq (3). This is a key assumption of the proposed numerical technique.

**Fig 4:**
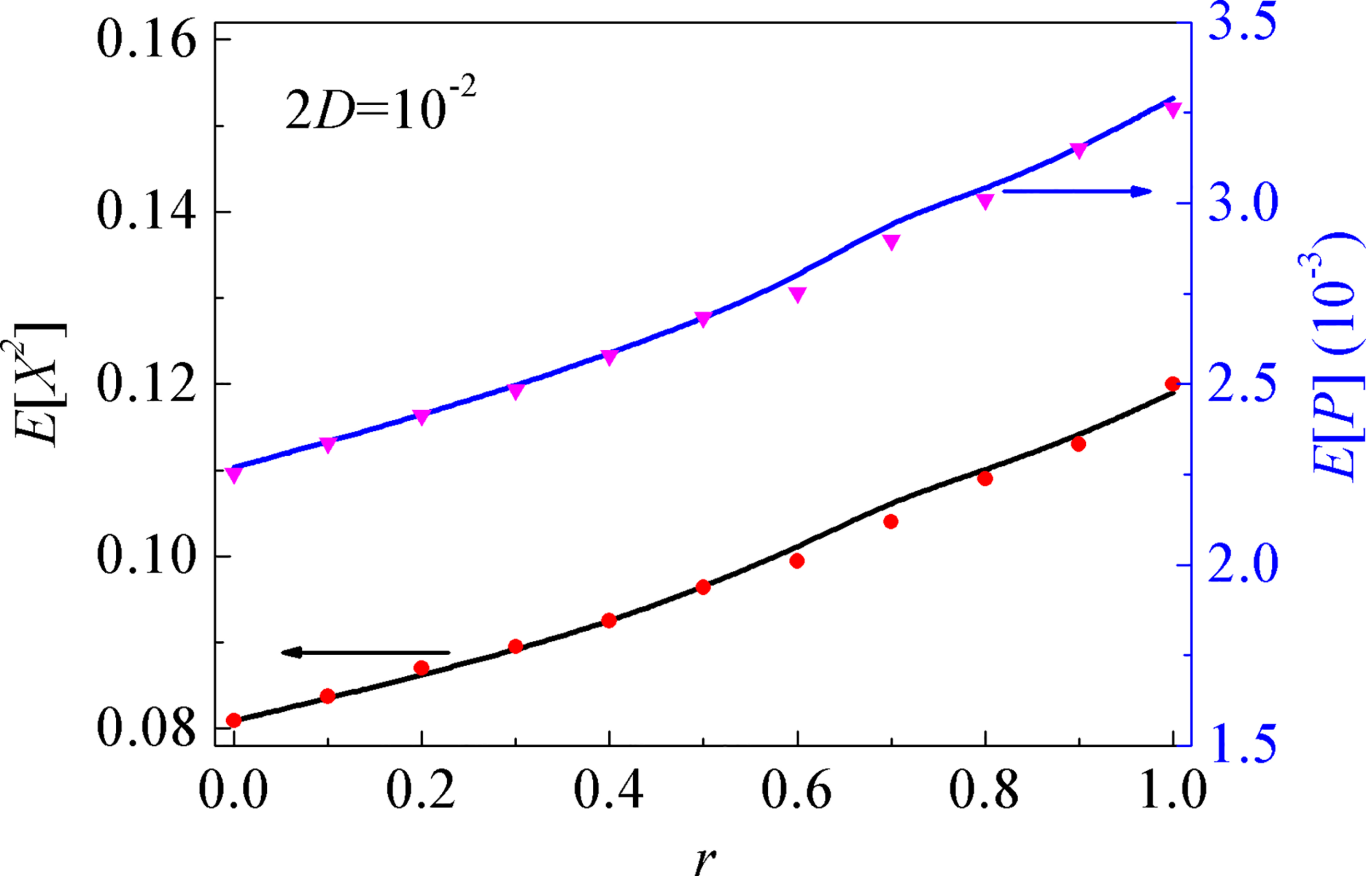
Variations of the mean-square displacement and output power with the adjustable stiffness. Solid lines: the proposed numerical results; symbols: MCS results.

Fig 4 depicts the dependences of the mean-square displacement *E*[*X*^2^] and the mean output power *E*[*P*] for energy harvesting on the adjustable parameter of the linear stiffness *r*. It is easily seen that the mean output power and the mean-square displacement increase with the parameter *r*, which due to the fact that the larger the adjusting stiffness parameter, the weaker the restoring force and the larger the displacement.

**Fig 5:**
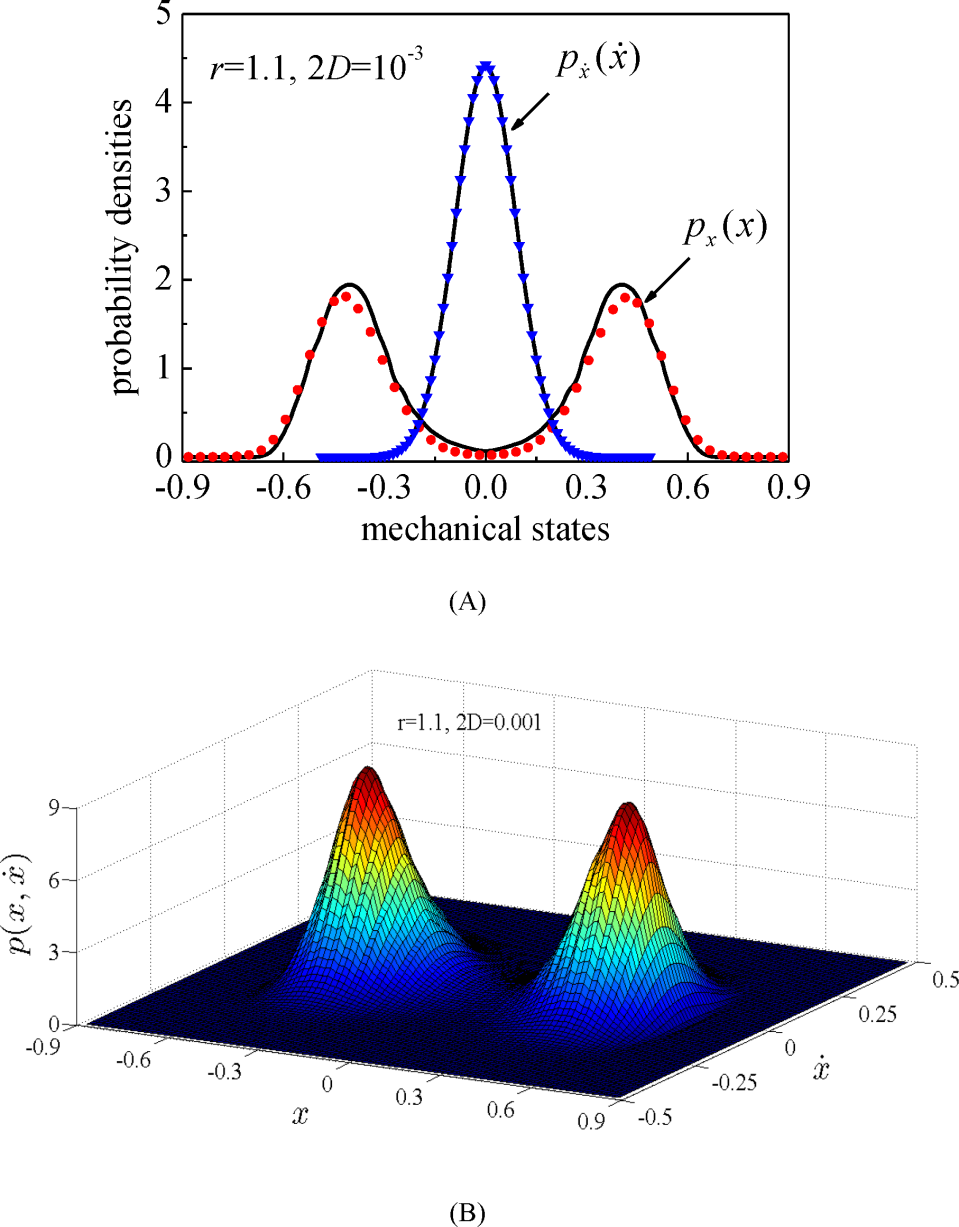
The stationary probability densities of system displacement and velocity for relatively small excitation. (A) The marginal probability density; (B) The joint probability density. Solid lines: the proposed numerical results; symbols: MCS results. (*r* = 1.1, 2*D* = 10^−3^).

**Fig 6:**
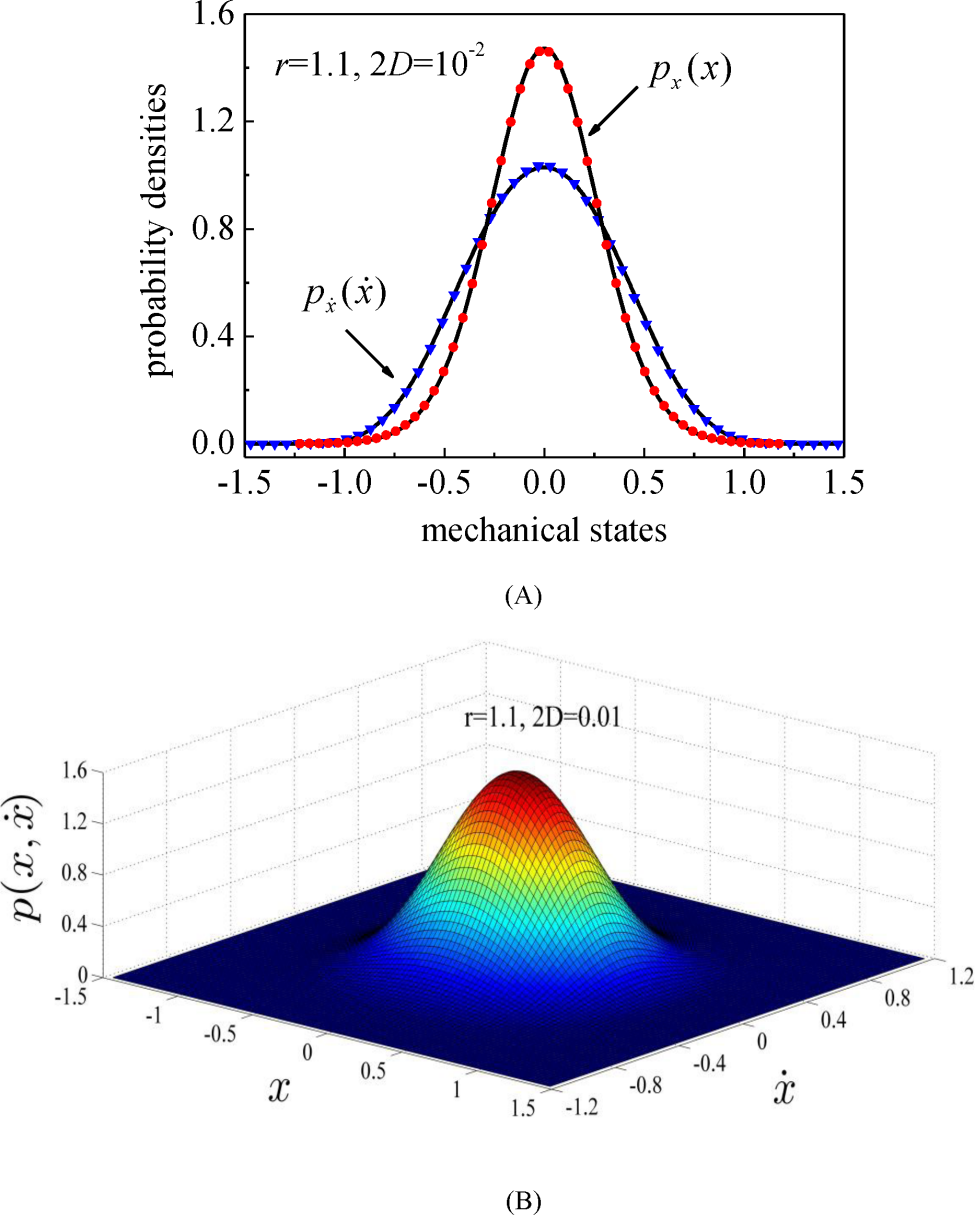
The stationary probability densities of system displacement and velocity for normal excitation. (A) The marginal probability density; (B) The joint probability density. Solid lines: the proposed numerical results; symbols: MCS results. (*r* = 1.1, 2*D* = 10^−2^).

For the situation of *r*>1 corresponding to the double-well system, the stationary marginal probability densities and the stationary joint probability density of system displacement and velocity are depicted in Figs 5 and 6 for two typical values of excitation intensity, 2*D* = 10^−3^ (relatively small excitation) and 2*D* = 10^−2^ (normal excitation), respectively. It is revealed in Fig 5 that the energy harvesting system possesses bistable potential shape while the motion frequently switches between two potential wells. Fig 6 depicts that the mechanical system possesses mono-stable potential shape, and the stationary probability density of system displacement does not display any bistable potential property. The random responses acquired by the splitting method meet well with those from MCS for the normal excitation (2*D* = 10^−2^), however, for the situation of relatively small excitation (2*D* = 10^−3^), the prediction results on the stationary probability density of system displacement are not very accurate.

**Fig 7:**
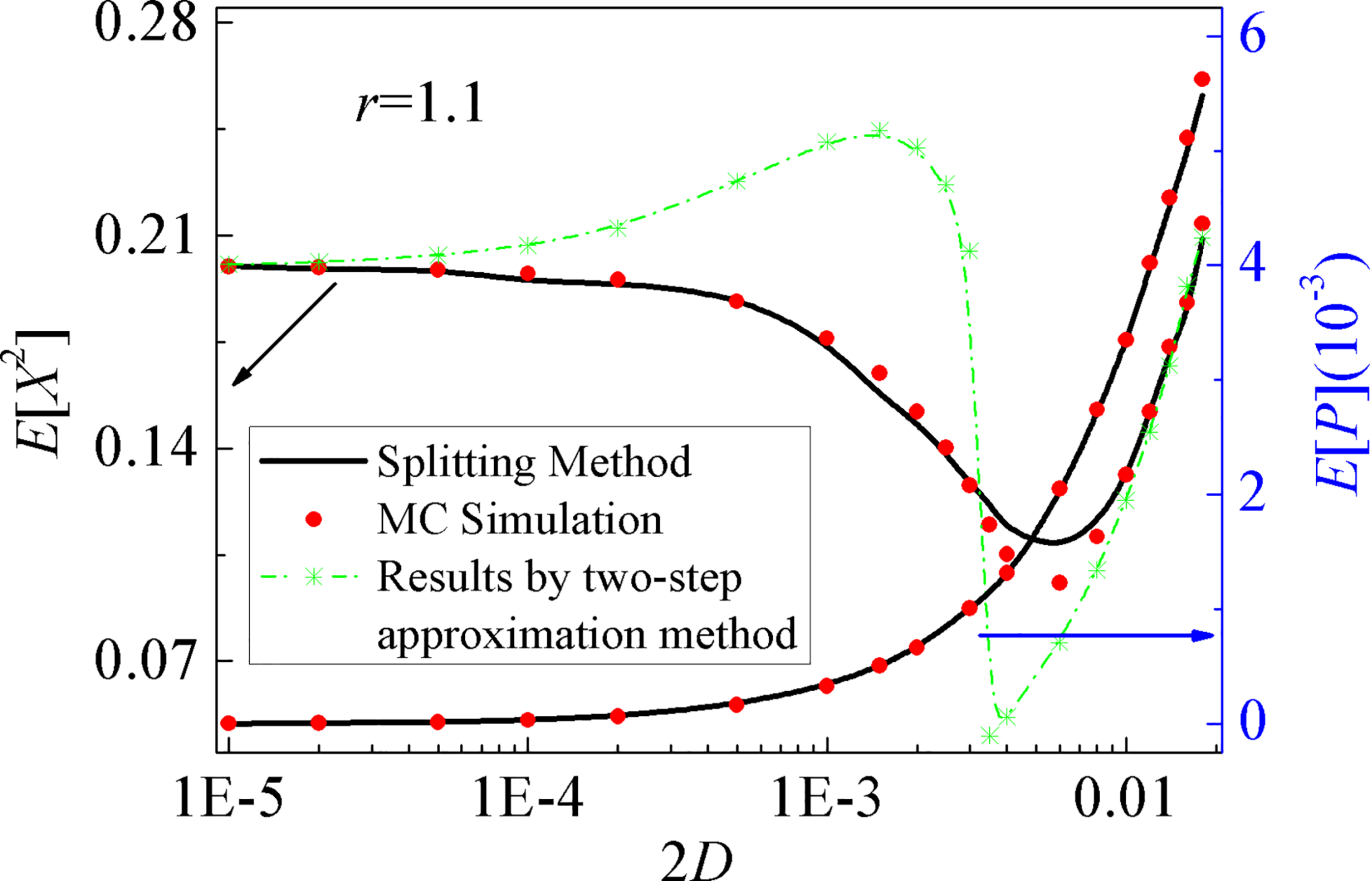
Variations of the mean-square displacement and the mean output power with the excitation intensity. Solid lines: the proposed numerical results; symbols: MCS results.

In the following section, the influences of the excitation intensity 2*D* on the mean-square displacement *E*[*X*^2^] and the mean output power *E*[*P*] for the bi-stable mechanical system are investigated and shown in Fig 7. With the increase of 2*D*, the mean-square displacement first decreases and then increases. The results obtained by the splitting method presented low accuracy for the excitation intensity remaining in some certain areas. For arbitrary excitation intensity in the concerned range, however, the splitting method gives high prediction accuracy on the mean output power. Furthermore, the value of the mean output power of the bi-stable system at the adjustable stiffness parameter *r* = 1.1 is obviously larger than that of the mono-stable system (0 < *r* < 1), which means in the random environment, the bi-stable harvesting system with elaborated design parameter can be superior to the mono-stable one.

### 3.2. Example 2: Duffing system with Dahl friction

**Fig 8:**
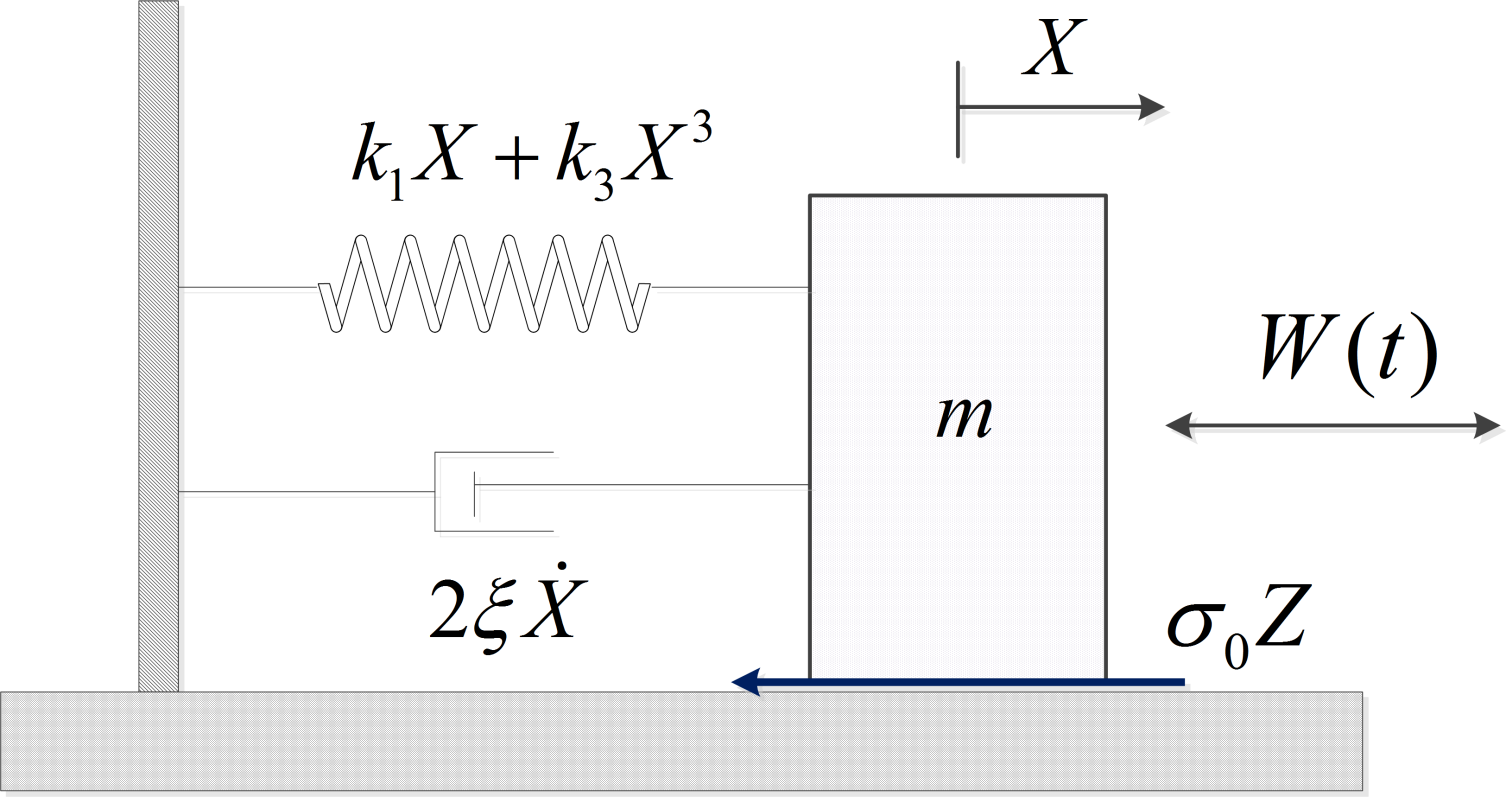
The schematic of Duffing system with Dahl friction.

The second example considers the following Duffing system with Dahl friction subjected to externally random excitation, as shown in Fig 8. The equations of motion of the system are written as

$$\begin{array}{l} \ddot{X}+2\xi \dot{X}+k_{1}X+k_{3}X^{3}+\sigma_{0}Z=W\left(t\right)\\ \dot{Z}=\dot{X}-\frac{\sigma_{0}\left|\dot{X}\right|}{f_{c}}Z \end{array}$$ (15)

where 2*ξ* is the viscous damping coefficient, *k*_1_ is the adjustable linear stiffness coefficient and *k*_3_ is the nonlinear stiffness coefficient representing the intensity of stiffness nonlinearity, *σ*_0_*Z* is the Dahl friction force, *f*_*c*_ is the Coulomb friction force, *W*(*t*) is Gaussian white noise with intensity 2*D*.

**Fig 9:**
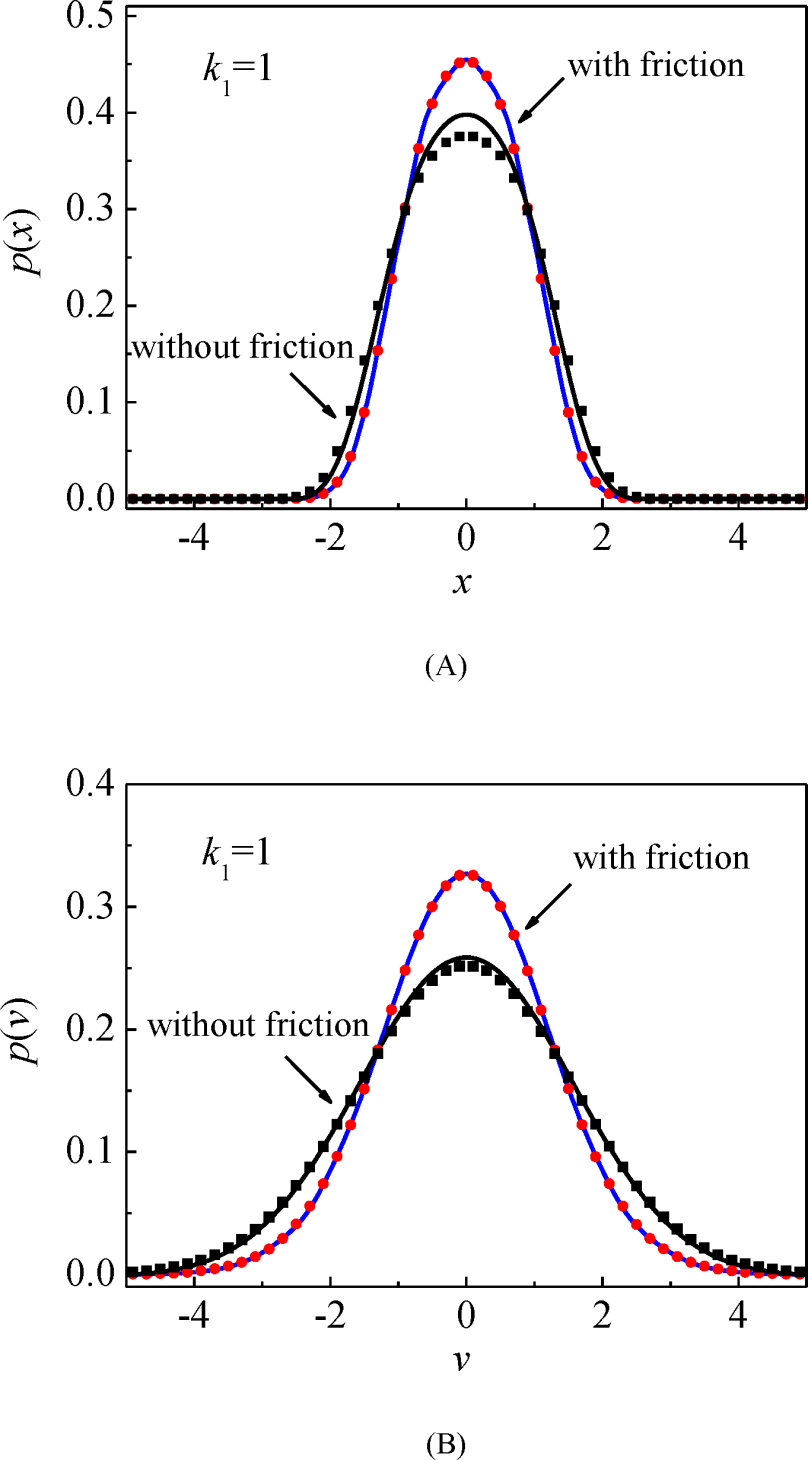
Stationary probability densities of system responses for mono-stable potential case. (A) Probability density of the displacement *x*; (B) Probability density of the velocity $\dot{x}$. Solid lines: the proposed numerical results; symbols: MCS results. (*k*_1_ = 1).

**Fig 10:**
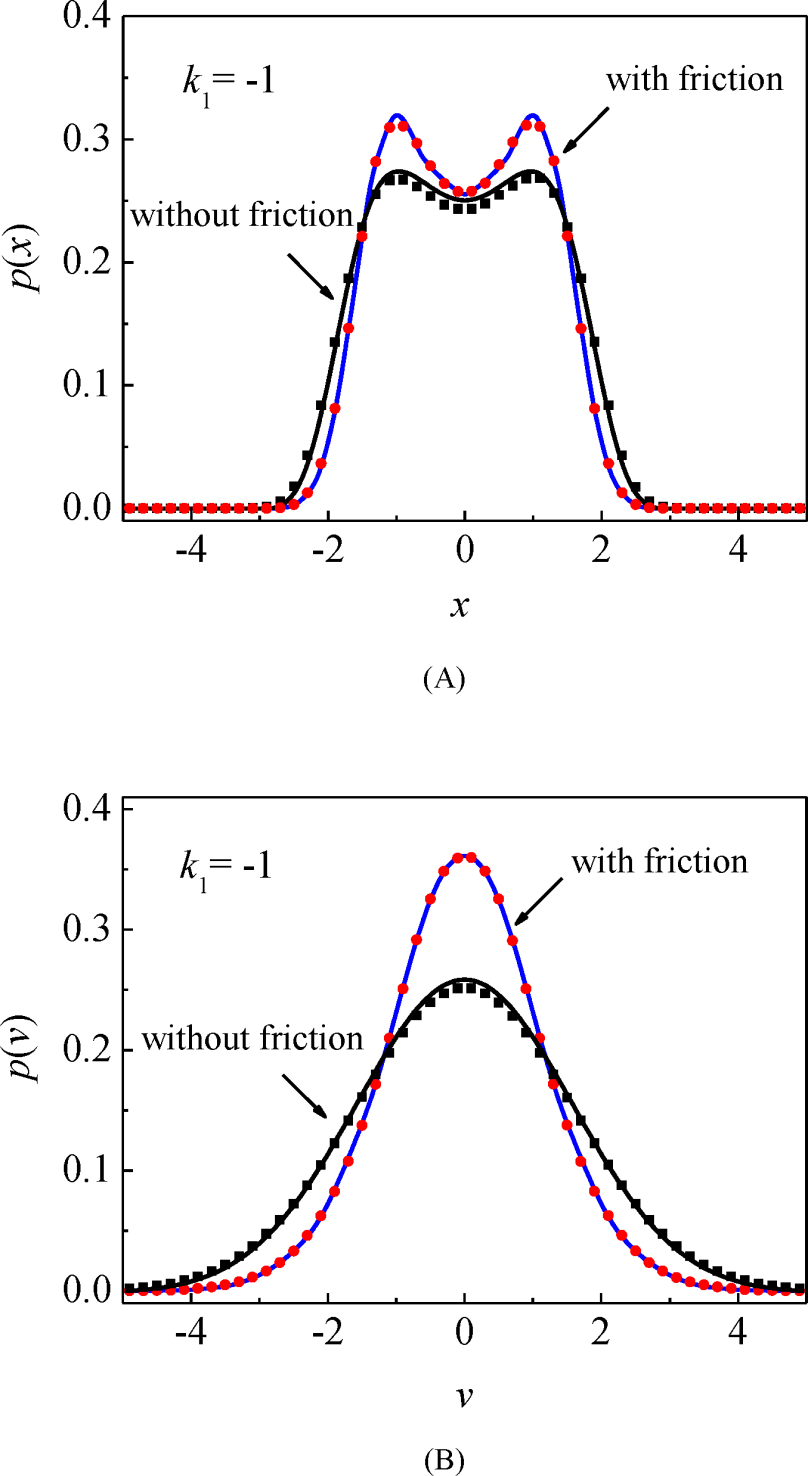
Stationary probability densities of system responses for bi-stable potential case. (A) Probability density of the displacement *x*; (B) Probability density of the velocity $\dot{x}$. Solid lines: the proposed numerical results; symbols: MCS results. (*k*_1_ = −1).

The random responses of the mechanical system (15) are investigated using the splitting method. Numerical results of the mono-stable potential case (*k*_1_ > 0) and bi-stable potential case (*k*_1_ < 0, *k*_3_ > 0) are shown to evaluate the effectiveness and accuracy of the proposed procedure. To illustrate the impact of the Dahl friction on the stochastic responses, the stationary probability densities of the corresponded friction-free systems are provided for comparison. The system parameters are set according to the study of Wang [37], the excitation intensity 2*D* = 0.1, the viscous damping coefficient *ξ* = 0.01, the nonlinear stiffness parameter *k*_3_ = 1, the Dahl friction force parameter *σ*_0_ = 0.06 and Coulomb friction force *f*_*c*_ = 0.05, except as specially supplied. We set the time step Δ*t =* 0.05 and the calculating time *T =* 200, and then acquire the stationary probability densities of system displacement and velocity as shown in Figs 9 and 10 for the mono-stable system and the bi-stable system, respectively. We also illustrate the accuracy of the splitting method via the results from MCS represented by the symbols. It is easily seen that the numerical solutions meet very well with the results from MCS for both the mono-stable and bi-stable systems.

**Fig 11:**
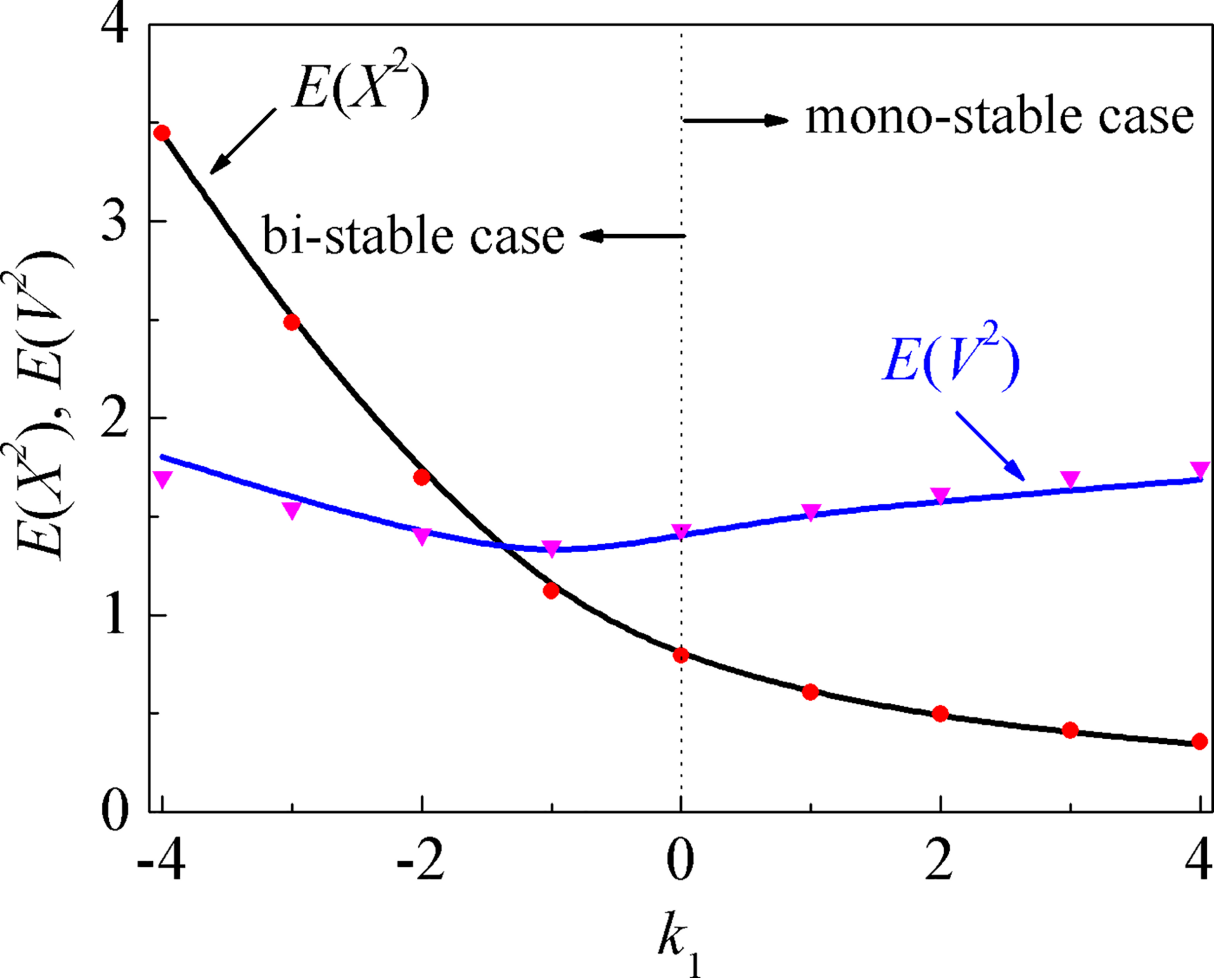
The dependence of the mean-square responses on the adjustable stiffness. Solid lines: the proposed numerical results; symbols: MCS results.

Fig 11 depicts the dependence of the mean-square responses on the adjustable linear stiffness parameter *k*_1_. It is obvious that the acquired results from the splitting method agree well with the MCS solutions for both the mono-stable case and bi-stable case. With the increase of *k*_1_, the Duffing system with Dahl friction turned from bi-stable case to mono-stable case, and switched at the point *k*_1_ = 0. The mean-square displacement decreases as *k*_1_ increases, however, the mean-square velocity first decreases and then increases.

**Fig 12:**
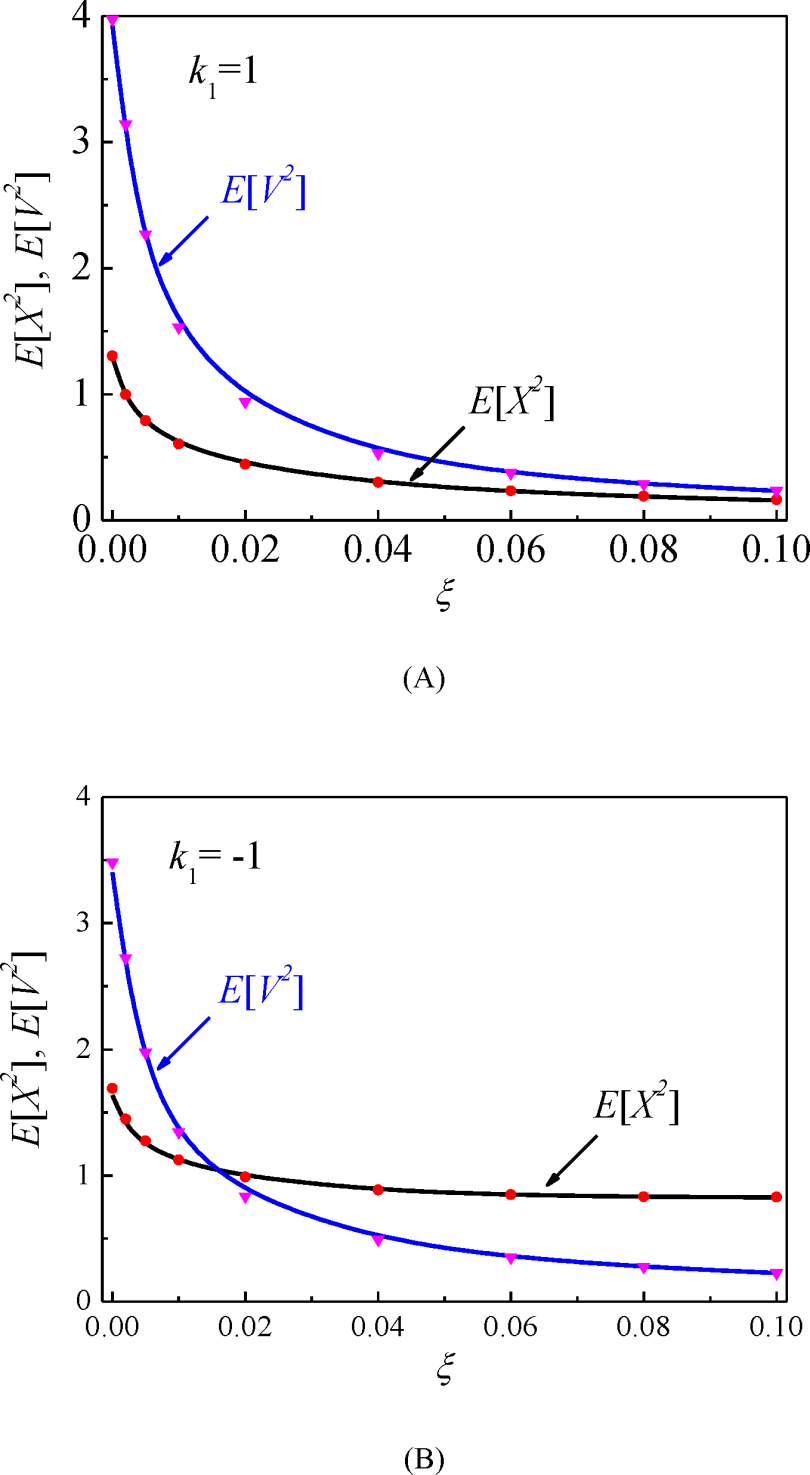
The dependence of the mean-square responses on the adjustable parameter of the linear viscous damping coefficient. Solid lines: the proposed numerical results; symbols: MCS results.

The relations of the mean-square displacement *E*[*X*^2^] and the mean-square velocity *E*[*V*^2^] to the linear viscous damping coefficient *ξ* are shown in Fig 12. For both the mono-stable (Fig 12A) and the bi-stable (Fig 12B) cases, the mean-square responses monotonically decrease with the viscous damping parameter *ξ*, and the rate of change reduces.

## 4. Conclusions

In this article, we investigate the random responses of the nonlinear vibration systems with adjustable stiffness property under Gaussian white noise excitation. Based on the original vibration systems rather than the equivalent systems, we propose a splitting method to solve the FPK equation associated with the Itô stochastic differential equation. We also obtain the stationary probability densities of system displacement and velocity as well as the mean-square displacement and the mean-square velocity.

To verify the accuracy and validate the applicability of the suggested approach, we present two classical nonlinear vibration systems with adjustable stiffness, i.e., the energy harvesting system and the Duffing system with Dahl friction as examples. For both the mono-stable and the bi-stable situation, we compare the results acquired by the splitting method with those from Monte-Carlo simulations. The agreement between the numerical results and MCS results validates the effectiveness of the proposed technique. The influences of system parameters are discussed in detail, including the excitation intensity, the viscous damping coefficient, the nonlinear stiffness coefficient, and especially the linear adjustable stiffness coefficient, on the stationary probability densities and the mean-square responses.

We conclude that the proposed procedure is more advanced than the stochastic averaging technique for the high excitation cases. We are currently working in addressing for engineering interests, such as higher-dimensional problems, strongly nonlinear systems and colored noise excitation situations, and we will report results in future publications.

## Supporting information

S1 File Operator splitting methods. (DOCX)

S2 File Chasing technique. (DOCX)

S3 File Selection of the optimal time step. (DOCX)

## References

[1] Kumar PR, Varaiya P (1986) Stochastic systems: estimation, identification and adaptive control. New Jersey: Prentice Hall.

[2] Besag J, Green P, Higdon D, Mengersen K (1995) Bayesian Computation and Stochastic Systems. Statistical Science 10: 3–41.

[3] Kulkarni VG (1995) Modeling and analysis of stochastic systems. London: Chapman & Hall.

[4] Anishchenko VS, Astakhov VV, Neiman AB, Vadivasova TE, Schimansky-Geier L (2002) Nonlinear Dynamics of Chaotic and Stochastic Systems. Berlin: Springer.

[5] Sunahara Y, Kabeuchi T, Asada Y, Aihara S, Kishino K (2003) On Stochastic Controllability for Nonlinear Systems. IEEE Transactions on Automatic Control 19: 49–54.

[6] Rudinger F (2007) Response spectral density for oscillators with nonlinear damping. J Eng Mech-ASCE 133: 278–289.

[7] Xu M, Wang Y, Jin XL, Huang ZL (2014) Random vibration with inelastic impact: equivalent nonlinearization technique. J Sound Vib 333: 189–199.

[8] Jin XL, Wang Y, Xu M, Huang ZL (2015) Semi-analytical solution of random response for nonlinear vibration energy harvesters. J Sound Vib 340: 267–282.

[9] Dimentberg MF, Gaidai O, Naess A (2009) Random vibrations with strongly inelastic impacts: Response PDF by the path integration method. Int J Non-linear Mech 44: 791–796.

[10] Narayanan S, Kumar P (2012) Numerical solutions of Fokker–Planck equation of nonlinear systems subjected to random and harmonic excitations. Probabilist Eng Mech 27: 35–46.

[11] Zhu HT, Duan LL (2016) Probabilistic solution of non-linear random ship roll motion by path integration. Int J Non-linear Mech 83: 1–8.

[12] Xu M, Wang Y, Jin XL, Huang ZL, Yu TX (2013) Random response of vibro-impact systems with inelastic contact. Int J Non-linear Mech 52: 26–31.

[13] Gu XD, Zhu WQ (2014) A stochastic averaging method for analyzing vibro-impact systems under Gaussian white noise excitations. J Sound Vib 333: 2632–2642.

[14] Anh ND, Hieu NN (2012) The Duffing oscillator under combined periodic and random excitations. Probabilist Eng Mech 30: 27–36.

[15] Jin XL, Wang Y, Huang ZL, Di Paola M (2014) Constructing transient response probability density of non-linear system through complex fractional moments. Int J Non-linear Mech 65: 253–259.

[16] Yang GD, Xu W, Gu XD, Huang DM (2016) Response analysis for a vibroimpact Duffing system with bilateral barriers under external and parametric Gaussian white noises. Chaos Soliton Fract 87: 125–135.

[17] Jiang WA, Chen LQ (2016) Stochastic averaging based on generalized harmonic functions for energy harvesting systems. J Sound Vib 377: 264–283.

[18] Rong HW, Wang XD, Xu W, Fang T (2010) Resonant response of a non-linear vibro-impact system to combined deterministic harmonic and random excitations. Int J Non-linear Mech 45: 474–481.

[19] Yang GD, Xu W, Jia WT, He MJ (2016) Random vibrations of Rayleigh vibroimpact oscillator under Parametric Poisson white noise. Commun Nonlinear Sci 33: 19–29.

[20] Dong Y, Han QL (2005) Delay-dependent exponential stability of stochastic systems with time-varying delay, nonlinearity, and Markovian switching. IEEE Transactions on Automatic Control 50: 217–222.

[21] Guo Y (2009) Mean square global asymptotic stability of stochastic recurrent neural networks with distributed delays. Applied Mathematics and Computation 215: 791–795.

[22] Guo Y (2013) Mean square exponential stability of stochastic delay cellular neural networks. Electronic Journal of Qualitative Theory of Differential Equations 34: 1–10.

[23] Guo Y (2017) Globally Robust Stability Analysis for Stochastic Cohen-Grossberg Neural Networks with Impulse Control and Time-Varying Delays. Ukrainian Mathematical Journal 69: 1220–1233.

[24] Guo Y, Xu C, Wu J (2017) Stability analysis of neutral stochastic delay differential equations by a generalization of Banach’s contraction principle. International Journal of Control 90: 1555–1560.

[25] Ghazizadeh S, Barbato M, Tubaldi E (2012) New Analytical Solution of the First-Passage Reliability Problem for Linear Oscillators. Journal of Engineering Mechanics 138: 695–706.

[26] Wang SL, Jin XL, Wang Y, Huang ZL (2014) Reliability evaluation and control for wideband noise-excited viscoelastic systems. Mechanics Research Communications 62: 57–65.

[27] Wang SL, Jin XL, Huang ZL, Cai GQ (2015) Break-out of dynamic balance of nonlinear ecosystems using first passage failure theory. Nonlinear Dynam 80: 1403–1411.

[28] Glowinski R, Pironneau O (1992) Finite element methods for Navier-Stokes equations. Annu Rev Fluid Mech 24: 167–204.

[29] Zorzano MP, Mais H, Vazquez L (1999) Numerical solution of two dimensional Fokker-Planck equations. Appl Math Comput 98: 109–117.

[30] Farago I (2005) Splitting methods for abstract Cauchy problems. Lect Notes Comput Sci 3401: 35–45.

[31] Cai GQ, Lin YK (2004) Probabilistic Structural Dynamics: Advanced Theory and Applications. New York: McGraw-Hill.

[32] Beeby SP, Tudor MJ, White NM (2006) Energy harvesting vibration sources for microsystems applications. Meas Sci Technol 17: R175–R195.

[33] Anton SR, Sodano HA (2007) A review of power harvesting using piezoelectric materials (2003–2006). Smart Mater Struct 16: R1–R21.

[34] Cottone F, Vocca H, Gammaitoni L (2009) Nonlinear Energy Harvesting. Phys Rev Lett 102: 4. 10.1103/PhysRevLett.102.080601 19257728

[35] Xu M, Jin XL, Wang Y, Huang ZL (2014) Stochastic averaging for nonlinear vibration energy harvesting system. Nonlinear Dynam 78: 1451–1459.

[36] Daqaq MF (2012) On intentional introduction of stiffness nonlinearities for energy harvesting under white Gaussian excitations. Nonlinear Dynam 69: 1063–1079.

[37] Wang Y, Luan XL, Jin XL, Huang ZL (2016) Random response evaluation of mono-stable and bi-stable Duffing systems with Dahl friction. Arch Appl Mech 86: 1827–1840.

