# Supplementary material for: Stochastic response analysis for nonlinear vibration systems with adjustable stiffness property under random excitation
Source: PLoS One. 2018 Aug 3;13(8):e0200922. doi: 10.1371/journal.pone.0200922 (PMC6075746; doi:10.1371/journal.pone.0200922)
Supplement: S1 File — (DOCX) [file pone.0200922.s001.docx]

**Operator Splitting Methods**

In this section, we address some basic notions about the splitting method. Firstly, consider an advective-diffusion equation of the form

$\frac{\partial u}{\partial t}=-A\frac{\partial u}{\partial x}+B\frac{\partial^{2}u}{\partial x^{2}}$ (S1.1)

where *A* and *B* are both constants. Then the equation can be differenced in the following schemes:

$\frac{u_{j}^{m+1}-u_{j}^{m}}{\Delta t}=-\frac{A}{2}\frac{u_{j+1}^{m}-u_{j-1}^{m}}{\Delta x}+B[\frac{u_{j+1}^{m}-2u_{j}^{m}+u_{j-1}^{m}}{(\Delta x)^{2}}]$ (S1.2)

$\frac{u_{j}^{m+1}-u_{j}^{m}}{\Delta t}=-\frac{A}{2}\frac{u_{j+1}^{m+1}-u_{j-1}^{m+1}}{\Delta x}+B[\frac{u_{j+1}^{m+1}-2u_{j}^{m+1}+u_{j-1}^{m+1}}{(\Delta x)^{2}}]$ (S1.3)

The former is the forward-time central-space (FTCS) scheme. The latter is exactly like the FTCS scheme (S1.2), except that the spatial derivatives on the right-hand side are evaluated at timestep *m*+1. Schemes with this character are called fully implicit or backward time, while the FTCS is called fully explicit. Simply form the average of the explicit and implicit schemes:

$\begin{matrix} \frac{u_{j}^{m+1}-u_{j}^{m}}{\Delta t}=-\frac{A}{4}(\frac{u_{j+1}^{m+1}-u_{j-1}^{m+1}}{\Delta x}+\frac{u_{j+1}^{m}-u_{j-1}^{m}}{\Delta x}) \\ +\frac{B}{2}[\frac{u_{j+1}^{m+1}-2u_{j}^{m+1}+u_{j-1}^{m+1}}{(\Delta x)^{2}}+\frac{u_{j+1}^{m}-2u_{j}^{m}+u_{j-1}^{m}}{(\Delta x)^{2}}] \end{matrix}$ (S1.4)

Here both the left- and right-hand sides are centered at timestep *m*+1/2, so Eq. (S1.4) combines the stability of an implicit method with the second-order accurate in both space and time.

Secondly, the basic idea of operator splitting method, which is also called time splitting or the method of fractional steps, is this: Consider an initial value equation of the form

(S1.5)

in which is some operator. Suppose that can be written as a linear sum of *n* pieces, which act additively on *p*,

(S1.6)

Suppose that for each of the pieces, we already know a differencing scheme for updating the variable *p* from timestep *m* to timestep *m*+1, valid if that piece of the operator were the only one on the right-hand side. These updatings are represented as

$\begin{matrix} p^{m+1}=F_{1}(p^{m},\Delta t) \\ p^{m+1}=F_{2}(p^{m},\Delta t) \\ ... \\ p^{m+1}=F_{n}(p^{m},\Delta t) \end{matrix}$ (S1.7)

where $F_{1}$ denotes an updating method that includes algebraically all the pieces of the total operator , but which is desirably stable only for the piece; likewise $F_{2}$, …,$F_{n}$. Then one form of operator splitting would be to get from *m* to *m*+1 by the following sequence of updatings:

$\begin{matrix} p^{m+1/n}=F_{1}(p^{m},\Delta t/n) \\ p^{m+2/n}=F_{2}(p^{m+1/n},\Delta t/n) \\ ... \\ p^{m+1}=F_{n}(p^{m+(n-1)/n},\Delta t/n) \end{matrix}$ (S1.8)

As each partial operation acts with all the terms of the original operator, the timestep for each fractional step in (S1.8) is now only $1/n$ of the full timestep.

Eq. (S1.8) is usually stable as a differencing scheme for the operator . Actually, it is often sufficient to have stable *F_i_*’s only for the operator pieces having the highest number of spatial derivatives, i.e., the other *F_i_*’s can be unstable and does not affect the stability of overall scheme. Then we turn our attention from initial value problems to boundary value problems.

Thirdly, we take for example the following *n*-dimensional FPK equation

$\frac{\partial p}{\partial t}=-A\frac{\partial p}{\partial x_{1}}-B\frac{\partial p}{\partial x_{2}}+C\frac{\partial^{2}p}{\partial x_{2}^{2}}+...-D\frac{\partial p}{\partial x_{n}}$ (S1.9)

Having chosen a splitting, we difference the time-dependent equation (S1.9) implicitly in *n* steps, a method of getting from $p^{m}$ to $p^{m+1}$ is

$\begin{matrix} \frac{p^{m+1/n}-p^{m}}{\Delta t}=-A\frac{\partial}{\partial x_{1}}(\frac{p^{m+1/n}+p^{m}}{2}) \\ \frac{p^{m+2/n}-p^{m+1/n}}{\Delta t}=-B\frac{\partial}{\partial x_{2}}(\frac{p^{m+2/n}+p^{m+1/n}}{2})+C\frac{\partial^{2}}{\partial x_{2}^{2}}(\frac{p^{m+2/n}+p^{m+1/n}}{2}) \\ ... \\ \frac{p^{m+1}-p^{m+(n-1)/n}}{\Delta t}=-D\frac{\partial}{\partial x_{n}}(\frac{p^{m+1}+p^{m+(n-1)/n}}{2}) \end{matrix}$ (S1.10)

The method is stable, second-order accurate in space and time and could be applied to simulate some more complicated cases: higher dimensional problem and time dependent coefficients.
