# Supplementary material for: Stochastic response analysis for nonlinear vibration systems with adjustable stiffness property under random excitation
Source: PLoS One. 2018 Aug 3;13(8):e0200922. doi: 10.1371/journal.pone.0200922 (PMC6075746; doi:10.1371/journal.pone.0200922)
Supplement: S2 File — (DOCX) [file pone.0200922.s002.docx]

**Chasing technique**

In this section, we briefly report the chasing technique for solving the space-domain finite difference discretization of Eqs. (7). Consider the following equation

$\frac{p^{m+1/3}-p^{m}}{\Delta t}=-\frac{\partial}{\partial X}(a_{1}\frac{p^{m+1/3}+p^{m}}{2})$ (S2.1)

As *X* is not included in *a*_1_, Eq. (S2.1) can be expressed as

$\frac{p_{i,j,k}^{m+1/3}-p_{i,j,k}^{m}}{\Delta t}=-\frac{a_{1}}{2}(\frac{p_{i+1,j,k}^{m+1/3}-p_{i-1,j,k}^{m+1/3}+p_{i+1,j,k}^{m}-p_{i-1,j,k}^{m}}{2\Delta X})$ (S2.2)

Set $s_{1}=\Delta t/\Delta X$ and $\alpha_{i}=-\frac{a_{1}s_{1}}{4},\beta_{i}=\eta_{i}=1,\gamma_{i}=\frac{a_{1}s_{1}}{4}$, we get the implicit difference equation of the form

$\alpha_{i}p_{i-1,j,k}^{m+1/3}+\beta_{i}p_{i,j,k}^{m+1/3}+\gamma_{i}p_{i+1,j,k}^{m+1/3}=-\alpha_{i}p_{i-1,j,k}^{m}+\eta_{i}p_{i,j,k}^{m}-\gamma_{i}p_{i+1,j,k}^{m}$ (S2.3)

After setting the value of function $p(x,y,z,t\left|x_{0}\right.,y_{0},z_{0},t_{0})$at *t* = *m*Δ*t*, we rewrite Eq. (S2.3) as

$\alpha_{i}p_{i-1}^{m+1/3}+\beta_{i}p_{i}^{m+1/3}+\gamma_{i}p_{i+1}^{m+1/3}=\delta_{i}$ (S2.4)

in which, $\delta_{i}=-\alpha_{i}p_{i-1}^{m}+\eta_{i}p_{i}^{m}-\gamma_{i}p_{i+1}^{m}$, the subscripts $j$ and $k$ are omitted as they have no influence on the partial derivatives of *X* (similarly hereinafter). We can also describe Eq. (S2.4) by the tridiagonal equations as

$A p=\delta$ (S2.5)

where $p=\left(p_{1}^{m+1/3},p_{2}^{m+1/3}, \cdots,p_{n}^{m+1/3}\right)^{T}$ and $\delta=\left(\delta_{1},\delta_{2}, \cdots,\delta_{n}\right)^{T}$, *A* is a tridiagonal matrix of the form

$A=\left(\begin{matrix} \beta_{1} \gamma_{1} 0 \cdots0 0 0 \\ \alpha_{2} \beta_{2} \gamma_{2} \cdots0 0 0 \\ 0 \alpha_{3} \beta_{3} \cdots0 0 0 \\ \vdots\vdots\vdots\vdots\vdots\vdots\\ 0 0 0 \cdots\alpha_{n-1} \beta_{n-1} \gamma_{n-1} \\ 0 0 0 \cdots0 \alpha_{n} \beta_{n} \end{matrix}\right)$ (S2.6)

which can be decomposed into $A =L U$ with

$L=\left(\begin{matrix} l_{1} 0 0 \cdots0 0 \\ \alpha_{2} l_{2} 0 \cdots0 0 \\ 0 \alpha_{3} l_{3} \cdots0 0 \\ \vdots\vdots\vdots\vdots\vdots\\ 0 0 0 \cdots l_{n-1} 0 \\ 0 0 0 \cdots\alpha_{n} l_{n} \end{matrix}\right)$ and $U=\left(\begin{matrix} 1 u_{1} 0 \cdots0 0 \\ 0 1 u_{2} \cdots0 0 \\ 0 0 1 \cdots0 0 \\ \vdots\vdots\vdots\vdots\vdots\\ 0 0 0 \cdots1 u_{n-1} \\ 0 0 0 \cdots0 1 \end{matrix}\right)$, where $\left\{\begin{matrix} l_{1}=\beta_{1},u_{i}=\gamma_{i}/l_{i} \\ l_{i+1}=\beta_{i+1}-\alpha_{i+1}u_{i} \\ i=1,2,...,n-1 \end{matrix}\right.$.

Then, we obtain the transition probability density at timestep *m*+1/3 via

$p=L^{-1}U^{-1}\delta$ (S2.7)

The abovementioned approach getting the values from one moment to the next through Eqs. (S2.3)-(S2.7) is called the chasing technique, which is widely used in solving implicit difference equations.

Analogously, we difference the second equation in Eqs. (7) in the following scheme:

$\begin{matrix} p^{m+2/3}-p^{m+1/3}=-\frac{\Delta t}{2}\frac{a_{2,j+1}p_{j+1}^{m+2/3}-a_{2,j-1}p_{j-1}^{m+2/3}+a_{2,j+1}p_{j+1}^{m+1/3}-a_{2,j-1}p_{j-1}^{m+1/3}}{2\Delta Y} \\ +\frac{\Delta t}{4}b_{22}\frac{p_{j+1}^{m+2/3}-2p_{j}^{m+2/3}+p_{j-1}^{m+2/3}+p_{j+1}^{m+1/3}-2p_{j}^{m+1/3}+p_{j-1}^{m+1/3}}{(\Delta Y)^{2}} \end{matrix}$ (S2.8)

Then, set$s_{2}=\Delta t/\Delta Y,r=\Delta t/(\Delta Y)^{2}$and$\alpha_{j}=-\frac{s_{2}}{4}a_{2,j-1}-\frac{r}{4}b_{22},\beta_{j}=1+\frac{r}{2}b_{22},\eta_{j}=1-\frac{r}{2}b_{22},$ $\gamma_{j}=\frac{s_{2}}{4}a_{2,j+1}-\frac{r}{4}b_{22}$, we get

$\alpha_{j}p_{j-1}^{m+2/3}+\beta_{j}p_{j}^{m+2/3}+\gamma_{j}p_{j+1}^{m+2/3}=\delta_{j}$ (S2.9)

where, $\delta_{j}=-\alpha_{j}p_{j-1}^{m+1/3}+\eta_{j}p_{j}^{m+1/3}-\gamma_{j}p_{j+1}^{m+1/3}$ are determined in the first step. Thus, we can easily obtain the transition probability density at timestep *m*+2/3.

Finally, the third equation in Eqs. (7) is considered and differenced as

$p^{m+1}-p^{m+2/3}=-\frac{\Delta t}{2}\frac{a_{3,k+1}p_{k+1}^{m+1}-a_{3,k-1}p_{k-1}^{m+1}+a_{3,k+1}p_{k+1}^{m+2/3}-a_{3,k-1}p_{k-1}^{m+2/3}}{2\Delta Z}$ (S2.10)

By setting $s_{3}=\Delta t/\Delta Z,\alpha_{k}=-\frac{s_{3}}{4}a_{3,k-1},\beta_{k}=\eta_{k}=1,\gamma_{k}=\frac{s_{3}}{4}a_{3,k+1}$, we get

$\alpha_{k}p_{k-1}^{m+1}+\beta_{k}p_{k}^{m+1}+\gamma_{k}p_{k+1}^{m+1}=\delta_{k}$ (S2.11)

in which, $\delta_{k}=-\alpha_{k}p_{k-1}^{m+2/3}+\eta_{k}p_{k}^{m+2/3}-\gamma_{k}p_{k+1}^{m+2/3}$ are determined by solving Eq. (S2.11). Thus, we obtain the PDF at *t* = (*m*+1)Δ*t*.
