# Supplementary material for: Stochastic response analysis for nonlinear vibration systems with adjustable stiffness property under random excitation
Source: PLoS One. 2018 Aug 3;13(8):e0200922. doi: 10.1371/journal.pone.0200922 (PMC6075746; doi:10.1371/journal.pone.0200922)
Supplement: S3 File — (DOCX) [file pone.0200922.s003.docx]

**Selection of the optimal time step**

In this section, we introduce the strategy for selecting the optimal time step. The following is its basic processes:

Firstly, large time steps lead to calculation divergence, while small ones lead to long calculation hours. Therefore, we usually assume a reasonable time step based on experience, for example Δ*t=*0.02 or Δ*t=*0.05.

Secondly, in order to ensure the convergence and stability of the proposed numerical procedure, taking Eq. (S2.1) to Eq. (S2.7) for example, the following conditions are generally satisfied:

(S3.1)

in which $\alpha_{i}=-a_{1}s_{1}/4,\beta_{i}=1,\gamma_{i}=a_{1}s_{1}/4,s_{1}=\Delta t/\Delta X$. Similarly, Eq. (S2.8) to Eq. (S2.9) must meet the requirements as

(S3.2)

where $\alpha_{j}=-\frac{s_{2}}{4}a_{2,j-1}-\frac{r}{4}b_{22},\beta_{j}=1+\frac{r}{2}b_{22},\gamma_{j}=\frac{s_{2}}{4}a_{2,j+1}-\frac{r}{4}b_{22},s_{2}=\Delta t/\Delta Y,r=\Delta t/(\Delta Y)^{2}$. Eqs (S3.1) and (S3.2) can be further applied to Eq. (S2.10) to Eq. (S2.11), and then we acquire the equation as

(S3.3)

where $s_{3}=\Delta t/\Delta Z,\alpha_{k}=-\frac{s_{3}}{4}a_{3,k-1},\beta_{k}=1,\gamma_{k}=\frac{s_{3}}{4}a_{3,k+1}$.

Thirdly, with system parameters given, the space step Δ*X* can be evaluated by Δ*X=X/nX*, where *nX* is determined by the calculating power of the computer and *X* is the approximate distribution of random response. Thus, we assess whether the earliest set time step 0.02 meets the applicable conditions (S3.1) to (S3.3) of the chasing technique. If Δ*t=*0.02 fulfills Eqs. (S3.1) to (S3.3), we gradually increase the total calculation time, and compare the calculated mean square values of random responses with MCS results to evaluate the convergence rate and stability of the numerical solutions. On the contrary, if the assumed Δ*t* does not meet the requirements, we gradually reduce the time step until it is appropriate.

Finally, we select the optimal time step by comparing the numerical solutions with the MCS results and considering the time-consuming of the numerical calculation. Shown in Table 1 is the influence of time step and calculating time on solving the mean square values of random responses, where the linear stiffness coefficient *r=*0, other parameters are the same as in Fig 4. *Error1* and *Error 2* are the relative error of the numerical results and the MCS results for the mean-square displacement *E*(*X*^2^) and the mean output power *E*(*P*), respectively.

**Table 1. The dependence of the mean-square responses on the time step and the calculating time.**

| Calculating time *T* | time step Δ*t=*0.02 | |  | time step Δ*t=*0.01 | | *Error1* (%) | | *Error2* (%) |
| --- | --- | --- | --- | --- | --- | --- | --- | --- |
|  | *E*(*X*^2^) *E*(*P*) | |  | *E*(*X*^2^) *E*(*P*) | |  |  |  |
| 50 | 0.115 | 0.00399 |  | 0.115 | 0.00399 | 42.2 | 77.3 | |
| 100 | 0.0850 | 0.00242 |  | 0.0850 | 0.00242 | 5.07 | 7.56 | |
| 150 | 0.0810 | 0.00227 |  | 0.0810 | 0.00227 | 0.123 | 0.889 | |
| 200 | 0.0805 | 0.00225 |  | 0.0805 | 0.00225 | 0.494 | 0.000 | |
| 250 | 0.0804 | 0.00225 |  | 0.0804 | 0.00225 | 0.618 | 0.000 | |
| 300 | 0.0804 | 0.00225 |  | 0.0804 | 0.00225 | 0.618 | 0.000 | |

Table 1 depicts that the time step variation does not affect the numerical solutions as long as the time step satisfies the applicable conditions of the chasing method. It is obvious that the mean square values of random responses tend to be stable and convergent as the calculation time *T* exceeds 150. Besides, the relative error of the results obtained by the proposed technique and the MCS results (*E*(*X*^2^)*=*0.0809, *E*(*P*)*=*0.00225) is less than 1%. Actually, we can only acquire the transient response but not the stationary random response under the situation of small *T*, which is the main reason for the relatively large error.

In conclusion, to ensure the convergence and stability of the presented method, we need to meet the following two points: one is the stochastic system enters the steady state; the other is that the chasing technique is applicable. The maximum time step that can satisfy these two points is the optimal time step, while the minimum T is the most suitable calculation time.
